# Supplementary material for: Prior Advanced Care Planning and Outcomes of Cardiopulmonary Resuscitation in the Emergency Department of a Comprehensive Cancer Center
Source: Cancers (Basel). 2024 Aug 13;16(16):2835. doi: 10.3390/cancers16162835 (PMC11353090; doi:10.3390/cancers16162835)
Supplement: Supplementary file 1 [file cancers-16-02835-s001.zip › cancers-3099722-supplementary.pdf]

Table S1. Demographics and clinical characteristics stratified by prior ACP or GOC documentation for the whole cohort undergoing resuscitation (N=100).

Variable	ACP or GOC documentation before the event		P
	No	Yes	
Total	56	44	
Age in years, mean ± SD	62.61 (13.40)	60.57 (10.74)	0.413
Sex			
Female	22 (39.3)	28 (63.6)	0.016
Male	34 (60.7)	16 (36.4)	
Race			
Asian	2 (3.6)	3 (6.8)	0.692
Black or African American	12 (21.4)	7 (15.9)	
White or Caucasian	36 (64.3)	27 (61.4)	
Other	6 (10.7)	7 (15.9)	
Ethnicity			
Hispanic or Latino	8 (14.3)	8 (18.2)	0.598
Not Hispanic or Latino	48 (85.7)	36 (81.8)	
CCI score, median [IQR]	6 [4–8]	8 [6–10]	0.006
Final cancer type			
Breast	1 (1.8)	3 (6.8)	0.367
Brain and spinal cord	0 (0.0)	2 (4.5)	
Endocrine	1 (1.8)	0 (0.0)	
Gastrointestinal	11 (19.6)	6 (13.6)	
Genitourinary	5 (8.9)	4 (9.1)	
Gynecologic	3 (5.4)	4 (9.1)	
Head and neck	9 (16.1)	4 (9.1)	
Leukemia	8 (14.3)	6 (13.6)	
Lung	11 (19.6)	5 (11.4)	

Other hematologic[a]	6 (10.7)	5 (11.4)	
Melanoma and other skin	1 (1.8)	1 (2.3)	
Sarcoma	0 (0.0)	3 (6.8)	
Other cancers	0 (0.0)	1 (2.3)	
Distant metastasis at the time of presentation			
Hematologic or CNS	14 (25.0)	13 (29.5)	0.377
No	17 (30.4)	8 (18.2)	
Yes	25 (44.6)	23 (52.3)	
Active cancer therapy			0.367
No	10 (17.9)	5 (11.4)	
Yes	46 (82.1)	39 (88.6)	
ROSC achieved			
No	20 (35.7)	13 (29.5)	0.515
Yes	36 (64.3)	31 (70.5)	

[a]Hematologic cancers besides leukemia.

Abbreviations: ACP, advanced care planning; GOC, goals of care; SD, standard deviation; CCI, Charlson comorbidity index; IQR, interquartile range; ROSC, return of spontaneous circulation
